# Analysis of HIF-1α expression and genetic polymorphisms in human clear cell renal cell carcinoma

**DOI:** 10.3389/pore.2023.1611444

**Published:** 2024-01-11

**Authors:** Daniela Vargova, Zuzana Kolková, Jan Dargaj, Lukas Bris, Jan Luptak, Zuzana Dankova, Sona Franova, Jan Svihra, Pavol Slávik, Martina Sutovska

**Affiliations:** ^1^ Department of Pharmacology, Jessenius Faculty of Medicine in Martin, Comenius University in Bratislava, Martin, Slovakia; ^2^ Biomedical Center Martin, Jessenius Faculty of Medicine in Martin, Comenius University in Bratislava, Martin, Slovakia; ^3^ Department of Urology, Jessenius Faculty of Medicine in Martin, Comenius University in Bratislava, and University Hospital Martin, Martin, Slovakia; ^4^ Department of Pathological Anatomy, Jessenius Faculty of Medicine in Martin, Comenius University in Bratislava, and University Hospital Martin, Martin, Slovakia

**Keywords:** HIF-1α, SNP, RCC, tumor, kidney

## Abstract

**Introduction:** Clear cell renal cell carcinoma (ccRCC) is mostly diagnosed incidentally and has relatively high recurrence rates. Alterations in VHL/HIF and mTOR pathways are commonly present in ccRCC. The present study attempted to identify potential diagnostic markers at the biochemical and molecular level.

**Methods:** In total, 54 subjects (36 patients with ccRCC and 18 cancer-free controls) were enrolled. ELISA was used to measure the levels of HIF-1α in the tumor and healthy kidney tissue. The association between five selected SNPs (rs779805, rs11549465, rs2057482, rs2295080 and rs701848) located in genes of pathologically relevant pathways (VHL/HIF and mTOR) and the risk of ccRCC in the Slovak cohort was studied using real-time PCR.

**Results:** Significant differences in HIF-1α tissue levels were observed between the tumor and healthy kidney tissue (*p* < 0.001). In the majority (69%) of cases, the levels of HIF-1α were higher in the kidney than in the tumor. Furthermore, the concentration of HIF-1α in the tumor showed a significant positive correlation with CCL3 and IL-1β (*p* (R2) 0.007 (0.47); *p* (R2) 0.011 (0.38). No relationship between intratumoral levels of HIF-1α and clinical tumor characteristics was observed. Rs11549465, rs2057482 in the *HIF1A* gene did not correlate with the expression of HIF-1α either in the tumor or in the normal kidney. None of the selected SNPs has influenced the susceptibility to ccRCC.

**Conclusion:** More research is neccesary to elucidate the role of HIF-1α in the pathogenesis of ccRCC and the association between selected SNPs and susceptibility to this cancer.

## Introduction

Renal cell carcinoma (RCC) is the most common type of primary kidney cancer with a clear cell variant (ccRCC) representing the majority of cases (70%–90%) [1]. The highest incidences of this cancer were recorded in North America and the Czech Republic [2]. RCC is mostly (60%) diagnosed incidentally and around 30% of patients have metastatic disease at the time of the diagnosis [3,4]. Five-year cancer specific survival (CSS) rates for patients with high grade (grades 3 and 4) tumors is significantly worse than for patients with low grade (grades 1 and 2) masses [5]. Furthermore, despite conventional therapy, a relatively high number of patients (20%–40%) experience recurrence [6]. Biological markers that would enable earlier diagnosis are therefore indispensable.

Various molecular mechanisms are involved in the pathogenesis of ccRCC. The most common alterations in this cancer are observed in the VHL/HIF pathway. Approximately 50%–60% of patients with sporadic ccRCC have some abnormality in the *VHL* gene [7]. Loss of the function of tumor suppressor protein VHL (pVHL) leads to accumulation and constitutive activation of hypoxia inducible factor 1α (HIF-1α) in cancer cells [8]. This transcription factor is responsible for the metabolic switch that allows survival of cells in the hypoxic environment. It induces the expression of genes involved in the Warburg effect (e.g., pyruvate dehydrogenase kinase) and angiogenesis (*VEGF, PDGF-BB*) [9–11]. Increased levels of HIF-1α are usually associated with a worse prognosis [12,13]. Additionally, HIF-1α is believed to play an important role in tumor-associated inflammatory signaling [14].

The mammalian target of rapamycin (mTOR) is a protein kinase that regulates cell growth and proliferation. Inappropriate activation of mTOR has been reported in RCC and in a variety of other cancers as well [15–17]. Once activated, mTOR affects the translation of oncogenic and angiogenic proteins (including HIF-1α), thus supporting cancer progression [7,18]. Aberrant mTOR signaling can result from genetic alterations in various components of the pathway (e.g., mTOR complexes or its upstream regulators PI3K and PTEN) [18].

Several single nucleotide polymorphisms (SNPs) were previously identified in the genes of the VHL/HIF and mTOR pathways. Among them, rs779805, rs11549465, rs2057482, rs2295080 and rs701848 were proposed to be potentially functional and responsible for the increased risk of various malignancies, including RCC [19–30].

The current study attempted to fulfill several objectives: 1.) analyze differences between the expression of HIF-1α in tumor and healthy renal tissue from patients diagnosed with ccRCC, 2.) analyze changes of HIF-1α tissue amount with respect to tumor nuclear grade (G) and tumor-node-metastasis (TNM) stage, 3.) determine the relationship between HIF-1α and cytokines in tumor tissue, and 4.) investigate whether any of the selected SNPs (rs779805, rs11549465, rs2057482, rs2295080 and rs701848) may serve as a potential diagnostic marker of ccRCC in Slovak cohort, by determining their association with the susceptibility to this cancer.

## Patients and methods

### Patients

In total, 54 subjects of Slovakian nationality participated in this study: 36 patients (28 men of 62.4 ± 8.3 years and 8 women of 63.6 ± 8.9 years) and 18 cancer-free controls (12 men of 65.1 ± 9.8 years and 6 women of 67 ± 5.9 years) aged 63.0 ± 8.4 and 66.0 ± 7.2 years, respectively. Both study groups were age-matched, as it is proved by statistically nonsignificant age difference between the two groups [*p* (t) 0.152 (1.45)]. Six female and 15 male patients had tumors of low grade (LG; grades 1 and 2). The other 15 subjects (3 women and 12 men) had high grade (HG; grades 3 and 4) tumors. Seven women and 19 men had the organ-confined (OC; pT1 and 2), while the other 2 females and 8 males had locally advanced (LA; pT3 and 4) tumors. The clinicopathological characteristics of patients study group is summarized in Supplementary Table S1. The inclusion criterium for the patients was histologically verified ccRCC that meets the criteria for surgical treatment. Any individual suffering from chronic disease of the urogenital system or any systemic diseases such as autoimmune, rheumatologic, endocrinne or severe cardiovascular disease were excluded from the study by taking a thorough medical history. The stage of the cancer was determined by computed tomography according to the World Health Organization 2016 classification criteria [31]. Tumor grade was determined by histopathological examination according to the WHO/ISUP (International Society of Urological Pathology) classification system [31]. Control subjects were recruited from individuals who underwent medical examination for non-malignant urological disease. They were not related to patients and had no history of any oncological disease. Both the healthy subjects and the patients provided 6 mL of peripheral venous blood. The blood was collected into EDTA tubes, promptly transported to the lab, and centrifuged at 3,000 rpm for 10 min. The buffy coat was then separated and stored at −80°C until further analysis. Tissue specimens (tumor- T and macroscopically normal kidney tissue- N) were obtained from patients during tumor removal performed at the Department of Urology of the Jessenius Faculty of Medicine in Martin and University Hospital Martin, Slovakia. Both ccRCC and healthy renal samples were removed from the same kidney, placed in sterile polypropylene tubes, and stored at −80°C until further analysis.

### DNA isolation and SNP genotyping

Genomic DNA was extracted from white blood cells using the DNeasy Blood & Tissue Kit (Qiagen). This simple and time-efficient procedure enables DNA isolation from fresh or frozen anticoagulated blood. First, blood samples were lysed with proteinase K in AL buffer (provided by the kit) during 10 min of incubation at 56°C. Ethanol (99.6%) was then added to the lysates and the mixture was loaded onto the DNeasy membrane in a mini spin column placed in a 2 mL collection tube and centrifugated at 8,000 rpm for 1 min. During this step, DNA bound to the DNeasy membrane, and excess fluid was removed. Subsequently, two additional centrifugation cycles were performed using AW1 and AW2 buffers (provided by the kit) to ensure the elimination of residual contaminants and increase the purity of the DNA. Finally, DNA was eluted in AE buffer (provided by the kit) and its concentration was evaluated using a NanoDropTM spectrophotometer (Thermo Scientific, USA) at 260 nm.

The extracted DNA was analyzed for individual genotypes using the TaqMan allelic discrimination method based on conventional real-time PCR. The genotyping reaction mixture consisted of genotyping TaqMan Master Mix, RNase-free water, and pre-designed Applied BiosystemsTM TaqMan® SNP genotyping assays. Genotypic detection was performed in the 7,500 Fast Real-Time PCR instrument (Applied BiosystemsTM, Thermofisher Scientific, USA). Characteristics of the selected SNPs are shown in Supplementary Table S2.

### Assessment of HIF-1α protein

HIF1α tissue levels were measured in the healthy kidney and ccRCC from 26 patients with the use of sandwich ELISA (LS-F4224). Clinical characteristics of these patients and all the raw data are described in the Supplementary Table S3. Supernatants used for the ELISA reaction were prepared according to the kit protocol as follows: 100 mg of tissue was washed with 0.02 M PBS (7.0–7.2 pH) to remove excess blood tissue and minced on ice. The samples were then placed into a tube containing 500 µL of 0.02 M PBS and homogenized on ice at 4,000 rpm (Homogenizer Stuart SHM2/Euro, Bibby Scientific, UK). The homogenates were subsequently subjected to a 3 freeze/thaw cycle and centrifuged at 7,170 rpm for 5 min at 4°C. Finally, the supernatant was collected, aliquoted, and frozen at −80°C until further analysis.

### Assessment of cytokine tissue levels

Cell cytokine tissue levels were determined using a commercially available kit Bio-PlexTM Human Cytokine Standard 27-Plex assay (Bio-Rad, Hercules, California, USA). This assay allowed us to detect 27 biologically relevant cell signaling molecules that are significantly involved in cancer biology: basic fibroblast growth factor (bFGF), CXCL11 (eotaxin), granulocyte macrophage colony stimulating factor (GM-CSF), granulocyte colony stimulating factor (G-CSF); interferon gamma (IFN-γ); interleukins 1β, 1Ra, 2, 4, 5, 6, 7, 8 (CXCL8), 9, 10, 12p70, 13, 15 and 17A; interferon gamma-induced protein 10 (IP-10 also known as CXCL10); monocyte chemoattractant protein 1 (MCP-1 also known as CCL2); macrophage inflammatory proteins 1 α and β (MIP-1α and MIP-1β also known as CCL3 and CCL4); platelet-derived growth factor-BB (PDGF-BB); RANTES (CCL5); tumor necrosis factor α (TNF-α), and vascular endothelial growth factor (VEGF). The biomarkers of interest were measured in each sample simultaneously during one reaction. This method combines two detection approaches: sandwich ELISA and flow cytometry. First, the target molecule reacts with the capture antibody coupled to magnetic beads, and then the unbound proteins are removed in series of washes. Next, biotinylated antibody is added, and a sandwich complex is formed. The streptavidin-phycoerythrin (SA-PE) conjugate is used to visualize the created complex. Identification and quantification of analytes are performed by two laser lights with different wavelengths (635 nm and 532) built in the Bio-Plex® 200 System reader. The final concentration of cytokines is proportional to the mean fluorescence intensity (MFI) and is expressed in pg/mL. To acquire standardized values, the tissue concentrations of HIF-1α and cytokines were divided by total tissue protein levels (mg/mL) measured by Bicinchoninic acid assay (BCA) kit (Thermofisher Scientific).

### Statistical analysis

A two-sample paired t-test was used to estimate the difference between HIF-1α expression in tumor (HIF-1α_TU_) and normal kidney tissue (HIF-1α_N_). Since the data were not trivial, the logarithmically transformed ratios of HIF-1α_TU_ and HIF-1α_N_ (log-T/N) of the patient samples were subjected to exploratory data analysis (EDA) and each observation was assigned to one of the two groups (Group 1 and Group 2) according to a higher posterior probability. The logarithmic ratios in each group were normally distributed. Group 1 was further analyzed using parametric unpaired t-tests (with Welch correction if necessary). The relationship between HIF-1α_TU_ and cytokines in tumor tissue was determined by a Pearson correlation coefficient. The final results were visualized using boxplots and scatter plots.

The null hypothesis of no association between SNP and ccRCC was tested by Fisher exacts test in general, dominant, recessive, and multiplicative models. Odds ratio (OR) with 95% confidence interval (95% CI) was computed for each model. Chi-square test was performed as well. The Hardy-Weinberg equilibrium test was tested in both patients and controls.

The data were explored and analyzed in R [1], ver. 4.0.5 and in GraphPad, ver. 8.0.1 software. The precision of the estimates was quantified by the 95% confidence limits. Dichotomization was performed using the cutoff *p*-value of 0.05.

## Results

### Genetic association study

We have performed a genetic association study (GAS) in five selected SNPs (rs779805 (*VHL*), rs2057482 (*HIF1A*), rs11549465 (*HIF1A*), rs2295080 (*MTOR*) and rs701848 (*PTEN*)) using various models (general, dominant, recessive, and multiplicative). Wild-type genotypes/alleles were used as reference. Genotype frequencies of all the SNPs were consistent with the Hardy-Weinberg equilibrium in both the patient and control group (at a level of significance 0.05). Interestingly, the distribution genotypes for rs2057482 and for rs11549465 in *HIF1A* were the same in all patients, but not in controls. We were unable to evaluate these two SNPs in a general or recessive model as the minor polymorphic genotype was present only in the control group. The results of GAS are summarized in Table 1. The raw data from this analysis are shown in Supplementary Tables S5, S6.

**TABLE 1:** The frequency of genotypes and alleles of selected gene polymorphisms in patients with ccRCC and controls.

Model	Genotype	Patients (N = 37)	Controls (N = 18)	OR	95% CI	*P*	*χ* ^*2*^ (*P*)
rs779805 (*VHL*) A > G							
G	AA	19 (53%)	9 (50%)				0.27 (0.87)
	AG	16 (44%)	8 (44.4%)	0.95	0.29–3.03	>0.99	
	GG	1 (3%)	1 (5.6%)	0.47	0.03–8.46	>0.99	
D	AA	19	9			
	AG + GG	17	9	0.90	0.29–2.78	>0.99
R	AA + AG	35	17			
	GG	1	1	0.48	0.03–8.25	>0.99
M	A	54	26			
	G	18	10	0.87	0.35–2.14	0.82
rs2057482 (*HIF1A*) C > T							
G	CC	27 (75%)	16 (88.9%)				
	CT	9 (25%)	1 (5.6%)	-	-	-	-
	TT	0 (0%)	1 (5.6%)				
D	CC	27	16				
	CT + TT	9	2	2.67	0.51–13.92	0.30
R	CC + CT	36	17			
	TT	0	1	-	-	-
M	C	63	33			
	T	9	3	1.57	0.39–6.20	0.75
rs11549465 (*HIF1A*) C > T							
G	CC	27 (75%)	14 (77.8%)			
	CT	9 (25%)	3 (16.7%)	-	-	-	-
	TT	0 (0%)	1 (5.6%)			
D	CC	27	14			
	CT + TT	9	4	1.17	0.31–4.47	>0.99
R	CC + CT	36	17			
	TT	0	1	-	-	*-*
M	C	63	31			
	T	9	5	0.89	0.27–2.87	>0.99
rs2295080 (*MTOR*) T > G							
G	TT	14 (39%)	7 (38.9%)				0.33 (0.85)
	TG	16 (44%)	9 (50.0%)	0.89	0.26–3.01	>0.99	
	GG	6 (17%)	2 (11.1%)	1.50	0.24–9.44	>0.99	
D	TT	14	7			
	TG + GG	22	11	1.00	0.31–3.19	>0.99
R	TT +TG	30	16			
	GG	6	2	1.60	0.29–8.86	0.70
M	T	44	23			
	G	28	13	1.13	0.49–2.58	0.84
rs701848 (*PTEN*) T > C							
G	TT	13 (36%)	3 (16.7%)				3.13 (0.21)
	TC	17 (47%)	13 (72.2%)	0.30	0.07–1.28	0.12	
	CC	6 (17%)	2 (11.1%)	0.69	0.09–5.29	>0.99	
D	TT	13	3			
	TC + CC	23	15	0.35	0.09–1.45	0.21
R	TT + TC	30	16			
	CC	6	2	1.60	0.29–8.86	0.70
M	T	43	19			
	C	29	17	0.75	0.34–1.69	0.54

The table shows a summary of the results of genetic association analysis for five selected SNPs in patients with clear cell renal cell carcinoma (ccRCC) and control subjects. The association was tested in four different genetic models: dominant (D), general (G), recessive (R) and multiplicative (M). The cut off value for the level of significance was 0.05. 95% CI, 95% confidence interval; OR, odds ratio; P, *p*-value of Fisher’s exact test; χ (P), Chi-squared test with *p*-value.

In all cases *p* values have reached extremely high levels. Therefore, it is not possible to draw any explicit conclusions from these results at the moment and further investigations are warranted. However, according to the odds ratios (ORs) of general and dominant models, we can hypothesize that one or two recessive alleles at rs779805 may possibly have protective character with regard to the risk of ccRCC [GG vs. AA: OR (95% CI) = 0.47 (0.03–8.46), *p* > 0.99; AG vs. AA: OR (95% CI) = 0.95 (0.29–3.03), *p* > 0.99; GG/AG vs. AA: OR (95% CI) = 0.90 (0.29–2.78), *p* > 0.99]. The results of recessive and multiplicative models appear to be in line with this hypothesis [GG vs. AA/AG: OR (95% CI) = 0.48 (0.03–8.25), *p* > 0.99; G vs. A: OR (95% CI) = 0.87 (0.35–2.14), *p* = 0.82]. The general and recessive models for rs2295080 suggest that individuals with two minor alleles could have one and a half times higher odds ratio of developing ccRCC than subjects with one or two major alleles [GG vs. TT: OR (95% CI) = 1.50 (0.24–9.44), *p* > 0.99; GG vs. TG/TT: OR (95% CI) = 1.60 (0.29–8.86), *p* = 0.70]. In other words, based on the OR values in general model, we can hypothesize that one minor allele probably may not be sufficient to increase the susceptibility to ccRCC [TG vs. TT: OR (95% CI) = 0.89 (0.26–3.01), *p* > 0.99]. As for rs701848, the results of individual models are contradictory. While recessive model indicates that individuals with two minor alleles have higher odds ratio of developing ccRCC [CC vs. TC/TT: OR (95% CI) = 1.60 (0.29–8.86), *p* = 0.70], neither general [CC vs. TT: OR (95% CI) = 0.69 (0.09–5.29), *p* > 0.99] nor multiplicative model agree with this [C vs. T: OR (95% CI) = 0.75 (0.34–1.69), *p* = 0.54].

### HIF-1α expression in ccRCC and kidney tissue

The results of our analysis have shown that two different groups of patients were present in our data set according to the means of log-HIF-1α_TU_ and HIF-1α_N_. Group 1 consisted of 18 patients (69% of all cases) and Group 2 comprised 8 patients (31%). The samples were further classified according to clinical characteristics of the tumor as LG or HG and OC or LA. In Group 2 there were 87.5% patients with HG tumors, while in Group 1 the majority (72%) of subjects had LG tumors. In Group 2, 37.5% of the patients had LA disease, while in Group 1 it was 28%.

**FIGURE 1:**
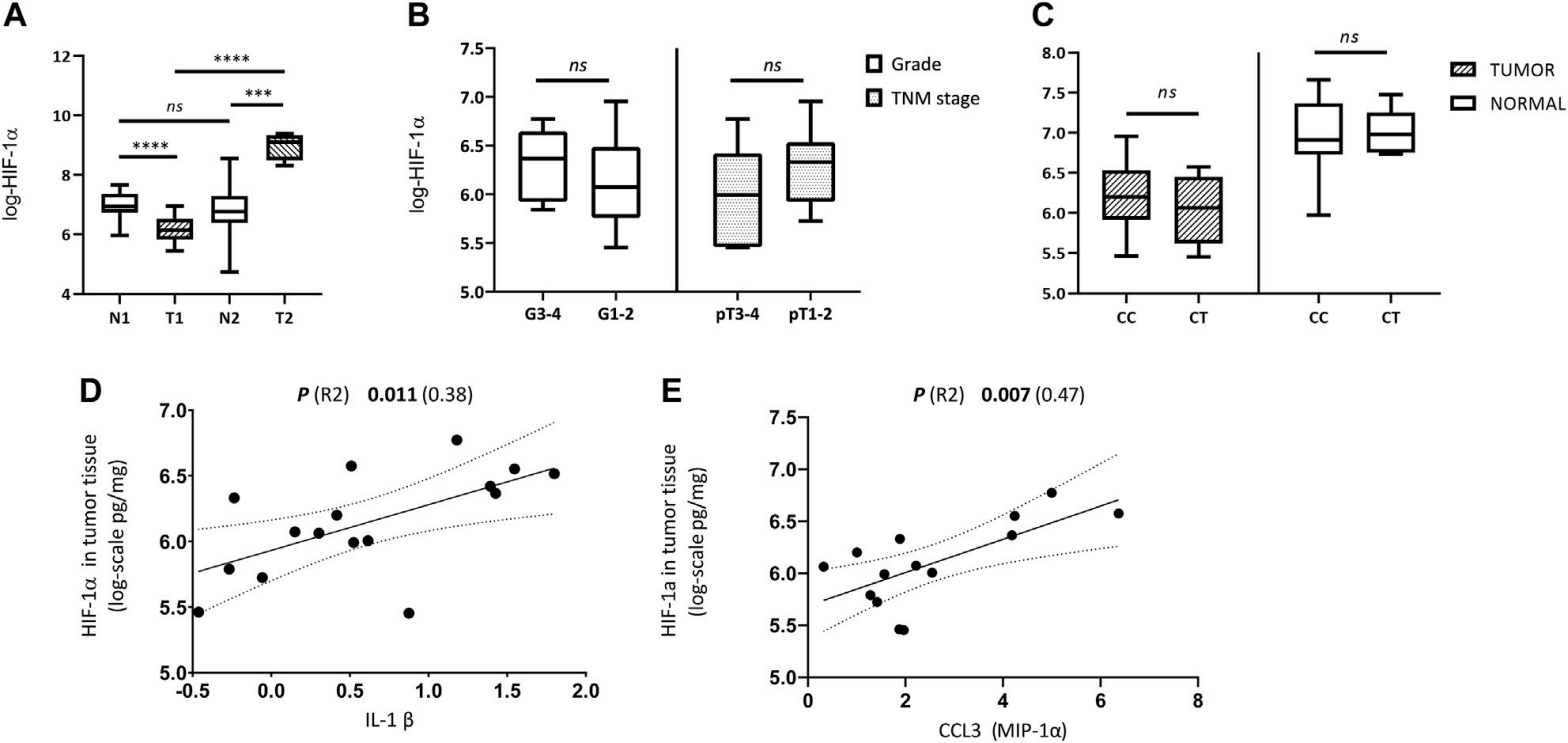
Comparison of HIF-1α tissue levels with regard to histological, clinical, genetic and immunological parameters. **(A)** Differences between HIF-1α expression in tumor and normal kidney tissue samples from patients with ccRCC in Group 1 (T1, N1) and Group 2 (T2, N2); **(B)** Differences in tumor tissue HIF-1α levels between high grade vs. low grade tumors (G3-4 vs. G1-2), and between locally advanced (LA) vs. organ-confined (OC) tumors (pT3 and 4 vs. pT1 and 2) of Group 1; **(C)** Difference in HIF-1α expression between tumor and normal kidney tissue of Group 1 according to genotypes (CC vs. CT) of rs2057482/rs11549465 in *HIF1A* gene; **(D)** Relationship between the amounts of HIF-1α and IL-1β in tumor tissue; **(E)** Relationship between the amounts of HIF-1α and CCL3 (MIP-1α) in tumor tissue. Concentration values of HIF-1α and cytokines used in the analyses were logarithmically transformed.

The means of log-HIF-1α_TU_ and HIF-1α_N_ values were compared between both groups using the unpaired t-test. The results have shown that the patients in Group 1 had higher levels of HIF-1α in normal tissue than in the tumor, while the individuals in Group 2 had exactly the opposite profile. The difference between the means of log-HIF-1α_TU_ and log-HIF-1α_N_ was statistically significant in both groups [*p* (t) < 0.0001 (5.82); *p* (t) = 0.0005 (5.28) respectively] (Table 2; Figure 1A). Furthermore, the tumor amount of HIF-1α in Group 1 was significantly lower compared to Group 2 [*p* (t) < 0.0001 (14.74)]. On the other hand, HIF-1α in normal tissue reached similar levels in both groups [*p* (t) = 0.64 (0.49)] (Table 2; Figure 1A). Since Group 1 was more numerous as to the samples, subsequent analyzes were performed only on this data set.

**TABLE 2:** Differences in HIF-1α concentration between two groups of patients and with regard to clinical and genetic parameters.

	Group 1	Group 2	T1 vs. T2	N1 vs. N2	LG vs. HG	OC vs. LA	CT vs. CC in tumor	CT vs. CC in normal tissue
N	(18, 18)	(8, 7)	(18, 7)	(18, 8)	(13, 5)	(13, 5)	(5, 13)	(5, 13)
*p-value*	<0.0001	0.0005	<0.0001	0.64	0.44	0.17	0.44	0.9
	****	***	****	*ns*	*ns*	*ns*	*ns*	*ns*
t statistics	5.82	5.28	14.74	0.49	0.78	1.42	0.79	0.13
Diff. of means (±SEM)	−0.81 (±0.14)	2.17 (±0.41)	2.78 (±0.19)	−0.19 (±0.39)	−0.18 (±0.23)	−0.31 (±0.22)	−0.18 (±0.23)	−0.03 (±0.22)
95% CI	−1.09 to −0.52	1.25 to 3.09	2.39 to 3.17	−1.09 to 0.71	−0.66 to 0.30	−0.77 to 0.15	−0.66 to 0.30	−0.49 to 0.44
R2	0.5	0.75	0.90	0.03	0.04	0.11	0.04	0.001

The Table shows a summary of unpaired t-test results comparing the means of logarithmically transformed HIF-1α concentration between different subpopulations: tumor vs. normal tissue in Group 1 and Group 2, tumor tissue of Group 1 (T1) vs. tumor tissue of Group 2 (T2), normal tissue of Group 1 (N1) vs. normal tissue of Group 2 (N2), Group 1 tumors of low grade (LG) vs. Group 1 tumors of high grade (HG), organ-confined tumors of Group 1 (OC) vs. locally advanced tumors of Group 1 (LA), Group 1 tumors with heterozygous genotype for rs11549465/rs2057482 vs. Group 1 tumors with homozygous genotype for rs11549465/rs2057482 (CT vs. CC in tumor) and finally Group 1 normal tissues with heterozygous genotype for rs11549465/rs2057482 vs. Group 1 normal tissues with homozygous genotype for rs11549465/rs2057482 (CT vs. CC in normal tissue); CI, confidence interval; diff, difference; N, number of data analyzed without outliers; ns, not significant; R2, R squared; SEM, standard error of the mean.

### Expression of HIF-1α and clinical tumor characteristics

We have examined tumor tissue expression of HIF-1α in the Group 1 according to clinical tumor characteristics using unpaired t-test. No significant differences in HIF-1α tumor tissue levels were observed between LG and HG [*p* (t) = 0.44 (0.78)] nor between OC and LA [p (t) = 0.17 (1.42)] (Table 2; Figure 1B).

### Effect of *HIF1A* polymorphisms on HIF-1α tumor and kidney expression

Next, we have analyzed whether the genetic status (genotype for rs2057482/rs11549465 in *HIF1A*) influences HIF-1α expression in either normal or tumor tissue. The mean value of log-HIF-1α in samples with the CC genotype was compared to log-HIF-1α in samples with the CT genotype. This was performed on tumor and normal tissue separately. No significant differences were detected in either case [*p* (t) = 0.44 (0.79); *p* (t) = 0.9 (0.13)] (Table 2; Figure 1C).

### Association between HIF-1α expression and cytokine amounts in ccRCC

Finally, the levels of 27 biologically relevant cytokines (listed in Materials and methods) were measured in tumor tissue of Group 1 and correlated with HIF-1α tumor tissue levels. CCL3 (MIP-1α) and IL-1β have shown a significant positive correlation with HIF-1α_TU_ [*p* (R-squared -R2) = 0.007 (0.47); *p* (R2) = 0.011 (0.38) respectively] (Table 3; Figures 1D, E). The raw data on cytokines used for this analysis are described in Supplementary Table S4 and were studied in more detail in our previous work [32].

**TABLE 3:** Association between HIF-1a and cytokines.

	Pearson r	95% CI	R2	*p*	Signif.
HIF-1α- IL-1β	0.62	0.18 to 0.85	0.38	0.011	*
HIF-1α-CCL3	0.69	0.24 to 0.89	0.47	0.007	**

The table shows the results of correlation analysis between HIF-1a and two cytokines (IL-1β and CCL3/MIP-1α). The data used in the analysis were logarithmically transformed. CI, confidence interval; R2, R squared; p, wo-tailed *p*-value; signif., significance with 0.05 cut-off.

## Discussion

HIF-1 is a ubiquitously expressed protein consisting of α and β subunits. Under normoxia, HIF-1α is hydroxylated and undergoes proteasomal degradation. Oxygen deficiency typical for solid tumors or abnormalities in the *VHL* gene that are often present in ccRCC, disrupt this process and lead to the accumulation of α subunit in the cytoplasm which is then translocated to the nucleus and initiates the transcription of its target genes. Increased expression of crucial proteins for angiogenesis, glucose metabolism, tumor growth and progression (VEGF, PDGF, EGFR, IGF, GLUT-1, CXCR, CAIX and XII), can contribute markedly to the tumorigenesis of RCC [11, 12]. The expression of HIF-1α was shown to correlate with the clear cell phenotype of RCC [10, 33]. The percentage of ccRCC specimens positive for high levels of HIF-1α reported in previous studies ranged from 17% to 97% [10]. The results of the present study have shown that the majority (69%) of the patients with ccRCC had significantly higher levels of HIF-1α in kidney than in tumor tissue. A possible explanation for these low HIF-1α levels in our ccRCC samples may be a deletion of 14q or alternative mRNA splicing leading to production aberrant HIF-1α isoforms [11, 34]. Alternatively, this observation may be related to the clinicopathological tumor profile of the samples. Tumors in which HIF-1α was significantly lower than in healthy adjacent kidney tissue were mostly (72%) organ-confined and low grade. Thus, we can hypothesize that HIF-1α levels depend on the disease stage. This, indeed, was confirmed in the study by Chen et al. (2020). These authors observed a positive correlation between *HIF1A* overexpression and the more aggressive tumor phenotype of various human cancers [35]. However, another study reported significantly lower HIF-1α levels in locally aggressive ccRCC tumors than in localized tumors [36]. Hoefflin et al. (2020) also suggested that the loss of *HIF1A,* which is supposed to cause a decrease in HIF-1α protein levels, is more frequent in high-grade and high-stage ccRCC tumors [9]. When we compared the HIF-1α tissue levels between LG and HG tumors, and between OC and LA tumors from patients with a low HIF-1α log-T/N ratio (Group 1), no significant differences were observed in either case. This corresponds to the study by Klatte et al. (2007) [12]. Neither these authors did find any association between HIF-1α tumor expression and clinicopathological tumor characteristics. Clearly, more research is necessary to explain these discrepancies and elucidate the role of HIF-1α in ccRCC pathogenesis and its relationship with disease progression. However, its involvement in the pathological processes of this tumor is supported by the fact that the normal kidney tissue of both groups we studied contained significantly different amounts of HIF-1α than tumor tissue.

HIF-1α is not only a master regulator of hypoxia, but it also participates in the regulation of innate and adaptive immune responses [37–40]. Inflammation is one of the prominent features of the tumor microenvironment (TME) [41]. The presence of highly complex crosstalk between the inflammatory and hypoxia pathways is supported by the fact that many of the key signaling molecules (cytokines and chemokines) are induced by low oxygen levels [42–44]. For example, in the context of rheumatoid arthritis, HIF-1α was shown to directly interfere with the production of TNF-α, IL-1β, IL-6 and IL-8 [45]. The role of HIF factors in sterile inflammation was also demonstrated in epithelial and fibroblast cancer cell lines [46]. In the present study, we found a statistically significant positive correlation between HIF-1α and CCL3, and between HIF-1α and IL-1β. Both CCL3 and IL-1β are known to participate in cancer pathogenesis [47,48]. Although data on the association between HIF-1α and these cytokines in the available literature are scarce, similar observations but in different circumstances have been reported. For example, inhibition of HIF-1α in multiple myeloma cells was shown to decrease CCL3 secretion [49]. In contrast, IL-1β appears to be rather upstream than downstream component of HIF-1α pathway. Jung et al., 2003 suggested that this interleukin may indirectly (through NF-κB and COX-2) mediate HIF-1α upregulation [50]. Clearly, further investigation is needed to determine the direction, character and consequences of the complex interaction between HIF-1α and these cytokines.

Rs11549465 (Pro582Ser/C1772T) and rs2057482 (C191T) are supposed to be functional polymorphisms. The former is found in the exon 12 of *HIF1A* and codes for the oxygen-dependent degradation domain of HIF-1α protein. The C > T substitution in rs11549465 is supposed to give rise to a molecule resistant to degradation which leads to increased transcriptional activity of HIF1 complex [19]. Rs2057482 is located in 3′UTR of *HIF1A.* There are several reports that hypothesized that the minor T allele in this polymorphism may decrease the *HIF1A* expression by creating a new microRNA binding site [22,51–54]. However, according to our data, neither the tumor nor the normal kidney HIF-1α tissue levels did not appear to be influenced by any of these two polymorphisms in *HIF1A*.

Regarding the results of our GAS, we could not interpret the association between the *HIF1A* genotypes (rs1149465, rs2057482) and the risk of developing ccRCC since no recessive homozygotes were present in the group of patients. The results of other studies on this SNP are inconsistent. The possible importance of variant alleles in rs11549465 with respect to RCC susceptibility was proposed in European patients [21]. Additionally, the meta-analysis by Wu et al. (2019) performed on Asian population revealed that the polymorphism rs2057482 was associated with decreased overall cancer risk [55]. On the other hand, no significant differences between RCC cases and controls in genotype frequencies for rs11549465 and rs2057482 were observed in the Chinese population [22]. These discrepancies may be ascribed to different ethnicities.

Polymorphism rs779805 is located within the *VHL* gene promoter, which is abundant in CpG islands and prone to methylation [56]. Therefore, we can hypothesize that A > G substitution may increase the probability of silencing of the *VHL* gene and subsequently increase the risk of developing ccRCC. Our results do not appear to support this assumption. Although the level of significance for either general or recessive model was far above the cut-off *p*-value, OR indicate that individuals carrying either one or two risk alleles could probably have lower risk of developing ccRCC. No significant differences in rs779805 genotypes between RCC cases and controls were reported also by Bensouilah et al. (2020) and Qin et al. (2012) [22, 29]. On the other hand, several other studies demonstrated a positive association between G allele at rs779805 and RCC risk [26–28].

The importance of mTOR pathway-related genes in RCC pathogenesis is well established [57–59]. Dysregulation of this pathway is usually associated with an aggressive tumor behavior [57]. Transcriptional activity of the *MTOR* gene can be altered in different ways. For example, a minor G allele at rs2295080 in the promoter region of *MTOR* was shown to be significantly associated with decreased mRNA levels of *MTOR* [23]. Consequently, several studies reported that Asian people carrying TG and TG/GG genotypes have a lower risk of urinary cancers (including RCC) than carriers of two dominant alleles [23,24,30]. Contrary to these, are the results of the present study. The relationship between this polymorphism and ccRCC risk was not significant, but the general and recessive models indicate that two minor alleles in this polymorphism could potentially increase the risk of ccRCC development.

Another frequently mutated gene in solid tumors, including RCC, is *PTEN* [60,61]. Loss of activity of this tumor suppressor gene is responsible for upregulation of the PI3K-Akt-mTOR pathway and eventually leads to enhanced cell growth and proliferation [62]. Rs701848 polymorphism located in 3′UTR of the PTEN gene is supposed to alter the binding affinity of miRNAs to this region and thus interfere with the process of gene splicing and protein expression [63,64]. Therefore, it is possible that the variant allele in rs701848 may influence cancer susceptibility. Indeed, a meta-analysis performed in Asian individuals showed that the recessive homozygous genotype (CC) at rs701848 is significantly associated with an increased risk of overall and urinary system cancer [25]. Regarding RCC, only a marginal association of rs701848 with this malignancy was reported in subjects from China [23]. We did not confirm any significant relationship between the minor allele or minor genotype in rs7018484 and the risk of ccRCC but there is an indication that minor allele could possibly have a protective character. However, more research is undoubtedly necessary to verify this.

To conclude, our study has shown significant differences in HIF-1α tissue expression levels between ccRCC and healthy kidney, with most cases having a significantly higher amount of HIF-1α in normal than in tumor tissue. Tumor tissue levels of CCL3 (MIP-1α) and IL-1β displayed significant positive correlation with the amount of HIF-1α in the tumor. None of the selected SNPs (rs779805, rs11549465, rs2057482, rs2295080 and rs701848) were associated with increased susceptibility to ccRCC. One of the major limitations of the study is a small data set. Therefore, the presented results should be considered as preliminary only.

## Data Availability

All relevant data is contained within the article and supplementary materials. Any further inquiries can be directed to corresponding authors.
