# Supplementary material for: Analysis of HIF-1α expression and genetic polymorphisms in human clear cell renal cell carcinoma
Source: Pathol Oncol Res. 2024 Jan 11;29:1611444. doi: 10.3389/pore.2023.1611444 (PMC10808674; doi:10.3389/pore.2023.1611444)
Supplement: Supplementary file 1 [file DataSheet1.pdf]

Table S1 *Clinicopathological characteristics of the group of patients*

**Group**	**Gender**	**N**	**Age mean ± SD (< 60; ≥ 60)**	**G**		**TNM**	
				< 3	≥ 3	< 3	≥ 3
	F	9	63.6 ± 8.9 (3; 5)	6	3	7	2
Patients (P)	M	27	62.4 ± 8.3 (9; 19)	15	12	19	8
	T	36	63.0 ± 8.4 (12; 24)	21	15	26	10

The table shows the number (N) and the average age of female (F) and male (M) patients, and the whole group (total, T) of patients. In each group (F, M and T) also the number of individuals aged less than 60 years (< 60) and individuals of 60 and more years old (> 60) is shown. Additionally, the distribution of females and males according to the histological tumor grade (G) and pathological disease stage (tumor-node-metastasis classification system; TNM) is shown. SD – standard deviation.

Table S2 *Basic characteristics of the selected SNPs*

**SNP ID**	**Gene** (Chr.)	**Context sequence** [VIC/FAM]	**Gene region**
**rs2295080**	*MTOR* (1)	GGGAGCGAGGGAAGGAGGGTTCCCA**[G>T]**CCCTGAGGACCAATCGACAGGTATA	promoter
**rs701848**	*PTEN* (10)	TGCTCCCCCGAGTTGGGACTAGGGC**[T>C]**TCAATTTCACTTCTTAAAAAAAATC	3'UTR
**rs2057482**	*HIF1A* (14)	CCTTTTTTTGGACACTGGTGGCTCA**[C>T]**TACCTAAAGCAGTCTATTTATATTT	3'UTR
**rs11549465**		GTTACGTTCCTTCGATCAGTTGTCA**[C>T]**CATTAGAAAGCAGTTCCGCAAGCCC	exon 12
**rs779805**	*VHL* (3)	GGCCTAGCCTCGCCTCCGTTACAAC**[A>G]**GCCTACGGTGCTGGAGGATCCTTCT	promoter

The table shows the identification number of the studied single nucleotide polymorphisms (SNP ID), gene title, the chromosome (Chr.) and the particular region of the gene in which they are located. The context sequence is given in the forward orientation and the nucleotides that bind fluorescent dyes (either VIC or FAM) are in square brackets and highlighted by blue color. HIF1A – hypoxia inducible factor 1A; MTOR – mammalian target of rapamycin; PTEN – phosphate and tensin homolog; 3'UTR – the three prime untranslated region; VHL – von Hippel Lindau.

Table S3 *Raw data from HIF-1α analysis and clinicopathological characteristics of the study group*

Patients Sample ID	Gender	Age	G	TNM	HIF-1α pg/ml		Proteins mg/ml		Standardized HIF-1α values pg/mg	
					Normal	ccRCC	Normal	ccRCC	Normal	ccRCC
94	Male	52	3	pT1bNxMx	7704.08	914.36	6.28	1.57	1226.02	581.53
95	Male	59	4	pT2aNxMx	18798.77	26758.19	3.63	2.35	5182.53	11379.59
96	Male	65	4	pT1bNxMx	6795.79	1642.74	4.26	2.43	1594.92	675.66
98	Male	71	3	pT1bNxMx	3988.97	13728.87	4.57	3.38	872.94	4058.81
99	Male	54	3	pT1bNxMx	5474.75	1018.15	5.49	2.96	997.47	344.06
101	Male	46	3	pT3aNxMx	2926.39	2830.69	3.26	3.24	897.73	873.39
103	Female	51	2	pT3aNxMx	3524.41	1716.61	3.28	3.99	1073.59	429.97
104	Male	69	1	pT1aNxMx	1883.21	1256.89	2.68	1.79	703.50	700.30
106	Male	68	4	pT3bNxMx	4553.69	30742.25	5.87	2.54	776.08	12099.35
107	Male	51	3	pT3aNxMx	4589.35	1764.01	5.52	4.35	830.72	405.89
108	Male	52	2	pT1aNxMx	4856.03	2419.96	4.31	3.38	1126.62	716.71
109	Male	74	2	pT1bNxMx	5230.26	4842.90	6.25	7.89	836.40	613.67
110	Male	70	1	pT1aNxMx	5227.73	2381.96	2.86	2.27	1830.29	1047.66
111	Male	61	4	pT3aNxMx	4031.66	32119.61	4.71	3.58	856.83	8974.99
113	Female	76	2	pT1bNxMx	6942.25	1734.62	7.93	5.30	875.18	326.99
114	Male	67	1	pT1bNxMx	543.16	1966.36	4.76	6.14	114.06	320.19
115	Female	63	4	pT2aNxMx	9723.41	34998.40	6.41	4.97	1516.79	7047.67
116	Female	66	1	pT1aNxMx	7293.69	2039.37	6.76	6.66	1078.44	306.22
117	Female	53	2	pT3aNxMx	7139.69	965.16	8.50	4.13	840.40	233.46
119	Female	67	3	pT1bNxMx	6740.48	21671.24	5.01	4.41	1346.24	4909.11

120	Male	65	2	pT4NxMx	3402.94	1453.00	3.39	6.17	1002.59	235.52
121	Female	62	1	pT1bNxMx	9950.67	2493.45	5.64	4.44	1763.73	561.90
122	Male	72	3	pT3aNxMx	1687.69	31182.37	3.12	3.22	540.77	9685.67
125	Male	61	2	pT1aNxMx	999.61	1644.84	2.55	3.79	391.59	434.27
126	Female	76	2	pT1aNxMx	6974.41	2627.17	4.43	5.33	1573.56	493.24
127	Male	61	1	pT2aNxMx	11839.84	1930.61	5.59	4.82	2118.52	400.37

The table shows the concentration of hypoxia inducible factor 1α (HIF-1α) in healthy kidney tissue (normal) and clear cell renal cell carcinoma tissue (ccRCC) was measured using ELISA method. BCA assay was used to determine the amount of total tissue proteins. G – histological grade of tumor; TNM – tumor-node-metastasis classification system.

Table S4 *Raw data from cytokines analysis of clear cell renal cell carcinoma tissue in Group 1*

**Patients ID**		**94**	**96**	**99**	**101**	**103**	**104**	**107**	**108**	**109**	**113**	**116**	**117**	**120**	**121**	**125**	**126**	**127**
**Logarithmically transformed standardized values of cytokines in tumor (WO)**	**bFGF**				5.08	5.50	6.40	5.85	4.81	7.15	4.75	5.31	5.51	4.78	4.02	4.83	5.75	5.66
	**G-CSF**	5.17	3.22		5.02	1.71	4.42	3.53	4.49	4.16	3.80	3.64	4.12	4.10	4.32	4.65	3.62	4.22
	**GM-CSF**	-0.75	-1.27		0.71	-1.09	1.40	-0.69	-1.05	-0.36	-0.57	-0.59	0.25	-0.01	-0.99	-0.31	0.07	0.30
	**IFN-γ**	1.83	-0.51		2.86	2.14	3.06	1.07	1.40	1.66	0.96	0.35	1.06	0.59	0.22	0.29	0.46	1.24
	**IL-1β**	1.43	1.80		1.18	0.30	1.55	0.61	0.51	1.39	-0.27	-0.06	0.87	-0.46	-0.24	0.15	0.42	0.52
	**IL-1ra**	6.82			6.19	4.78	5.90	5.91	5.80	5.82	5.25	5.02	5.32	5.90	5.13	5.24	5.67	5.13
	**IL-2**	1.29	0.52		0.99	-0.22	1.78	0.22	0.29	0.96	0.21	0.48	1.31	0.05	-1.02	0.14	0.84	0.87
	**IL-4**	1.04	0.00		-0.10	-1.15	0.42	-0.40	-0.55	-0.59	-0.25	-0.03	0.89	-0.43	-0.57	-0.13	0.44	0.55
	**IL-5**	3.39	2.46	5.98	3.55	0.78	3.89	1.48	0.91	1.54	2.39	2.73	3.53	2.22	0.98	2.29	2.89	3.35
	**IL-6**	2.11	0.78	4.78	0.65	0.66	1.74	1.84	3.87	3.56	1.67	0.92	2.02	0.65	-0.86	0.66	0.33	1.15
	**IL-7**	2.37	1.23		2.53	2.32		1.39	0.59	0.01	0.27	0.12	0.51	2.20	1.21	2.34	0.79	1.41
	**IL-8**	2.25	1.68	5.20	2.30	2.23	3.64	1.57	3.08	2.68	2.25	1.95	1.54	3.03	1.47	2.81	3.60	2.03
	**IL-9**	3.74	2.03		4.17	3.06	3.80	3.37	3.11	2.94	3.33	3.08	3.48	4.12	3.74	4.05	3.83	3.66
	**IL-10**	0.34	-0.96	3.11	0.87		0.49	-0.78	-0.95	-1.46	-1.55	-1.76	-0.79	-1.47	-2.22	-2.06	-1.07	-0.94
	**IL-12**	0.51	-0.69	4.21	2.34	0.19	3.38	-0.11	-0.66	-0.07	0.57	1.27	1.56	1.59	0.73	1.30	1.38	2.04
	**IL-13**	-0.54	-2.48	3.63	2.01	-0.46	0.96	-1.26	-1.39	-1.84	-1.13	-1.53	-0.83	0.73	-1.25	-0.98	0.37	0.52
	**IL-15**	4.25	3.07	7.18					2.13	2.85	2.59	2.74	3.47	4.14			3.31	3.40
	**IL-17**	2.72	1.68		1.56	0.62	2.19	0.90	0.89	1.38	0.79	1.27	2.13	0.76	0.40	0.88	1.53	1.77

	**CXCL10**	8.34			10.94	5.05	8.06	7.49	6.27	6.44	6.62	6.11	7.44	5.51	4.98	6.13	5.42	6.72
	**CCL2**	3.91		7.57	4.43	2.95	5.81	4.53	5.09	5.14	4.20	2.96	4.24	4.99	4.20	4.06	3.91	4.98
	**CCL3**	4.18			5.00	0.32	4.24	2.54	6.37		1.28	1.42	1.96	1.87	1.88	2.22	1.00	1.57
	**CCL4**	6.10	3.67		5.87	2.97	4.97	4.02	4.39	5.40	3.25	3.39	4.05	4.32	3.46	3.87	3.53	3.68
	**CCL5**						8.20				6.26	6.51	6.80	6.94	5.36	6.08	6.77	5.58
	**TNFα**	3.64	3.34		3.13	1.82	2.82	1.97	2.09	2.48	2.13	2.18	2.97	2.65	1.77	2.44	2.67	2.78
	**VEGF**	1.16	6.31	8.33	6.55	2.03	4.91			1.35	5.17	3.58	4.95	4.04	4.51	4.09	4.20	5.22
	**Eotaxin**	0.27	-0.81	5.51	1.21	-0.63	2.09	2.50	2.41	0.02	0.54	0.06	0.67	0.18	1.51	0.51	0.61	1.09
	**PDGF-BB**	4.53	4.68	7.23	3.65	2.35	7.17	2.33	2.04	2.51	3.21	3.11	4.69	3.29	2.11	3.53	3.87	3.08

The table shows logarithmically transformed standardized concentrations (originally expressed in pg/mg) of 27 cytokines measured in clear cell renal cell carcinoma tissue from 17 patients of Group 1. WO – without otuliers.

Table S5 *Raw data from genotyping analysis in patients and clinicopathological characteristics of the study group*

**Patients Sample ID**	**Clinicopathological parameters**				**Genotypes of selected polymorphisms**				
	Gender	Age	G	TNM	rs779805 (*VHL A>G*)	rs2057482 (*HIF1A C>T*)	rs11549465 (*HIF1A C>T*)	rs2295080 (*MTOR T>G*)	rs701848 (*PTEN T>C*)
94	Male	52	3	pT1bNxMx	AG	CC	CC	TG	TC
95	Male	59	4	pT2aNxMx	AG	CC	CC	TG	CC
96	Male	65	4	pT1bNxMx	AG	CC	CC	TG	TC
98	Male	71	3	pT1bNxMx	AG	CT	CT	GG	TC
99	Male	54	3	pT1bNxMx	AA	CC	CC	TT	TT
101	Male	46	3	pT3aNxMx	AA	CC	CC	TT	TC
103	Female	51	2	pT3aNxMx	AA	CT	CT	TT	TC
104	Male	69	1	pT1aNxMx	AG	CC	CC	TG	TC
106	Male	68	4	pT3bNxMx	AG	CC	CC	TG	TT
107	Male	51	3	pT3aNxMx	AA	CC	CC	TT	TC
108	Male	52	2	pT1aNxMx	AA	CT	CT	TT	TC
109	Male	74	2	pT1bNxMx	AA	CC	CC	TT	TC
110	Male	70	1	pT1aNxMx	AA	CC	CC	GG	TT
111	Male	61	4	pT3aNxMx	AA	CC	CC	TG	CC
113	Female	76	2	pT1bNxMx	AA	CT	CT	TG	TC
114	Male	67	1	pT1bNxMx	AA	CC	CC	TG	CC
115	Female	63	4	pT2aNxMx	AG	CC	CC	TG	TC
116	Female	66	1	pT1aNxMx	AA	CC	CC	TT	CC
117	Female	53	2	pT3aNxMx	AG	CT	CT	GG	TT
119	Female	67	3	pT1bNxMx	AG	CC	CC	TT	TT
120	Male	65	2	pT4NxMx	AA	CC	CC	TT	TT
121	Female	62	1	pT1bNxMx	GG	CT	CT	TT	TT

122	Male	72	3	pT3aNxMx	AG	CT	CT	TG	TT
125	Male	61	2	pT1aNxMx	AG	CC	CC	TT	TT
126	Female	76	2	pT1aNxMx	AA	CC	CC	TG	TT
127	Male	61	1	pT2aNxMx	AA	CC	CC	TT	TT
128	Male	63	2	pT1bNxMx	AG	CC	CC	GG	TT
130	Female	58	3	pT1bNxMx	AG	CC	CC	GT	TC
131	Male	74	2	pT1bNxMx	AA	CC	CC	GT	TC
132	Male	49	4	pT3aNxMx	AA	CC	CC	GG	TC
134	Male	69	1	pT1bNxMx	AA	CC	CC	TT	TC
136	Male	62	2	pT2aNxMx	AG	CC	CC	GT	CC
139	Male	54	2	pT1bNxMx	AG	C	CC	GG	CC
142	Male	63	1	pT1bNxMx	AA	CT	CT	TT	TC
143	Male	76	3	pT1aNxMx	AA	CC	CC	GT	TT
145	Male	58	2	pT3aNxMx	AG	CT	CT	GT	TC

The table shows a distribution of genotypes of five selected polymorphisms tested in group of 36 patients. G – histological tumor grade; HIF1A – hypoxia inducible factor 1A; MTOR – mammalian target of rapamycin; PTEN – phosphate and tensin homolog; TNM – tumor-node-metastasis classification system; VHL – von Hippel Lindau.

Table S6 *Raw data from genotyping analysis in control subjects and characteristics of the study group*

Controls Sample ID	Gender	Age	Genotypes of selected polymorphisms				
			rs779805 (*VHL A>G*)	rs2057482 (*HIF1A C>T*)	rs11549465 (*HIF1A C>T*)	rs2295080 (*MTOR T>G*)	rs701848 (*PTEN T>C*)
1	Male	69	AA	CC	CT	TT	CC
2	Male	66	AG	CC	CC	GT	TC
3	Male	53	AG	CC	CT	GT	TC
4	Male	66	AA	CC	CC	TT	TC
5	Male	71	AG	CC	CC	GT	CC
6	Male	66	AA	CC	CC	GT	TC
7	Male	63	AA	TT	TT	GT	TT
8	Male	65	AG	CC	CC	TT	TC
9	Male	71	AG	CC	CC	TT	TC
10	Male	69	AA	CC	CC	GG	TT
11	Male	56	AA	CC	CC	GT	TC
12	Male	71	AG	CC	CC	TT	TC
13	Female	67	AG	CC	CC	TT	TC
14	Female	63	AA	CC	CC	GG	TC
15	Female	67	AA	CC	CC	GT	TC
16	Female	52	AG	CC	CC	TT	TC
17	Female	82	AA	CC	CC	GT	TT
18	Female	71	GG	CT	CT	GT	TC

The table shows a distribution of genotypes of five selected polymorphisms tested in group of 18 control subjects without any oncological disease. HIF1A – hypoxia inducible factor 1A; MTOR – mammalian target of rapamycin; PTEN – phosphate and tensin homolog; VHL – von Hippel Lindau.
